# Supplementary material for: Are carbon emissions trading and green financial instruments synergistic? -Comprehensive quantitative research based on content analysis
Source: PLoS One. 2024 Mar 7;19(3):e0298601. doi: 10.1371/journal.pone.0298601 (PMC10919601; doi:10.1371/journal.pone.0298601)
Supplement: S1 Data — (ZIP) [file pone.0298601.s001.zip › Data/地级市Z_F工作报告.zip!/┴╔─■/┼╠╜⌡╩╨/└┤╫╘╣1⁄2╓┌║┼í╛┬φ┐╦╩2╛▌═°í┐_2016.docx]

各位代表：

　　现在，我代表市政府，向大会报告工作，请予审议，并请各位政协委员和其他列席同志提出意见。

　　一、2015年和“十二五”工作回顾

过去的一年，面对异常严峻复杂的经济形势，在市委的正确领导下，我们全面贯彻党的十八届三中、四中、五中全会精神，深入落实习近平总书记系列重要讲话精神和长春座谈会 “四个着力”总要求，认真贯彻落实省委、省政府“四个驱动、六个新的经济增长点”决策部署，攻坚克难，开拓创新，全市经济社会保持平稳健康发展。

　　主要经济指标稳步增长。扣除不可比因素，预计地区生产总值实现1100亿元，同比增长7%；固定资产投资983亿元，增长8%；一般公共预算收入95亿元，增长1%；社会消费品零售总额345亿元，增长14%。多数指标增速高于全省平均水平，持续走在全省前列。城镇登记失业率2.48%，低于全省平均水平。居民消费价格同比持平。

　　发展质量和效益有效提升。城镇常住居民人均可支配收入33000元，增长7%；农村常住居民人均可支配收入13860元，增长9%，总量继续保持全省前列。高新技术产品增加值占规上工业比重达到25%。万元GDP能耗、主要污染物排放均有所下降。

　　一批事关全局的重大项目实现重要突破。盘锦港晋升国家一类口岸，享有了与国内知名港口同等的开放地位。总投资924亿元的兵器精细化工及原料工程获得核准，30万吨原油码头配套工程环评获批，盘锦建设世界级石化及精细化工产业基地迈出关键一步。总投资850亿元的忠旺铝材全产业链加工基地启动实施。北燃公司获批700万吨进口原油使用资质，成为东北地区首家取得该资质的民营企业。总投资150亿元的中储粮东北综合产业基地开工建设。这些重大项目的落地，极大提振了全市人民的信心。

　　新的增长动力加快孕育。服务业占比提高到35%，消费拉动作用进一步增强。自主创新能力明显提高，突破30多项关键技术，22项科技项目转化落地。小巨人企业、楼宇经济蓬勃发展，新产业、新业态、新模式不断涌现。

　　一年来，我们围绕全面建成小康社会，以项目制的工作方式狠抓658项重点任务落实，各项工作取得显著成效。

　　（一）持之以恒抓项目稳投资，努力增强发展后劲。我们把扩大有效投资作为转型发展的关键，坚定不移抓招商、抓项目，以优质项目提升经济效益。

　　深入贯彻落实国家支持东北振兴等重大举措。争取到6项国家级省级试点示范政策平台，大洼县被列入国家中小城市综合改革试点，撤县改区及国有农场职工住宅用地流转等改革有序推进。争取各类专项资金突破100亿元。央企沪企民企合作等重大项目取得积极进展。

　　强力推进存量项目建设，加大跟踪服务力度，全市亿元以上产业项目开复工412项，同比增加39项；竣工133项，增加31项。北燃芳烃合成、汇福粮油等40个投资10亿元以上重大项目加快建设，为稳定经济增长提供了有力支撑。

　　把招商引资作为扩大有效投资的重要举措，面向长三角、珠三角、京津冀、台湾等重点地区高强度走出去、请进来，实施北京、昆山、德国驻地招商，全市签约注册优质项目170项，实际利用外资2.5亿美元。德国赢创、巴斯夫、沙特阿美等项目深入推进。

　　加强基础设施建设，实施滨海公路、辽河生态文明示范路等12项重点交通工程和76项城建工程，加快建设12项水利工程，有效拉动了投资。

（二）建设辽东湾创业新城，创造开放发展新优势。我们举全市之力推进新区建设，在建区10周年的重要时间节点，推动新区发展迈上了新台阶，走在全省国家级开发区前列。

　　不断完善港口与口岸功能，10个5万吨级以上泊位加快建设，沈盘疏港通道竣工通车，集疏运体系更加完善，港口吞吐能力达到5000万吨。联检大厦投入使用，口岸查验机构全面升级，保税物流中心建设快速推进。主动融入“一带一路”战略，盘满欧集装箱国际班列开通运营，盘锦成为辽宁三条通往欧洲国际大通道的重要节点。

　　加快壮大产业规模，宝来石化、和运集团搬迁扩产积极推进，联成化学、益海嘉里、二界沟渔雁小镇、金帛海岸等项目加快建设。全年实施亿元以上项目72项，引进32项。

　　深入推进创业新城建设，公共服务体系、生活服务设施和创业孵化功能不断完善，石化、粮食两大交易中心加快推进，复制创新上海自贸区等76项改革事项，新区人气商气加速集聚。

（三）全力转方式调结构，提高发展质量和效益。我们顺应市场需求和科技进步新变化，加快转变发展方式，提高供给体系质量和效率，促进三次产业向中高端迈进。

　　工业经济提质增效。落实《中国制造2025》，支持企业技术改造和设备更新，北沥润滑油加氢等41项技改项目顺利推进。以“两化”融合为重点，实施工业企业自动化水平提升专项行动，加快发展智能制造。海工装备成为国家重点支持的产业集群。落实企业帮扶责任制，辽河油田、华锦集团、辽河石化等大企业稳产增效，为全市稳增长作出了突出贡献。规模以上工业增加值增速位居全省首位。

　　旅游业迅速发展壮大。把旅游业作为一项大产业来谋划，用大旅游的理念促进三次产业转型升级。27个重点旅游项目加快建设，景区品位和功能实现质的飞跃，红海滩旅游度假区成为国家级生态旅游示范区。成功举办红海滩国际马拉松赛，加快塑造辽河口渔家菜等文化品牌，推出湿地游、乡村游、文化游等特色旅游产品，四季旅游活动精彩纷呈，主要景区接待游客增长75%。在中央和省级主流媒体全方位扩大宣传，城市知名度大幅提升。红海滩成为比肩西湖、黄山等著名景区的旅游热点，成为盘锦对话世界的窗口。

　　服务业优质高效发展。大力发展电子商务，在全省率先创建市级电商公共服务平台，促进互联网向生产、流通、消费等各领域拓展，批发零售、住宿餐饮、物流配送方式加快向线上线下交易转变。省级服务业集聚区发展到7个，主营业务收入同比增长20%。3个省级物流园区加快建设。盘锦成为我省农产品现代流通体系和肉菜流通可追溯体系建设试点市。推出30条促进房地产业健康发展的新政策，举办大型房交会，商品房销售额增速保持全省前三名。

　　农业现代化扎实推进。坚持走质量效益型发展道路，积极扩大绿色有机无公害农作物种植，认养农业发展到4.3万亩。启动建设2条现代农业示范带。着力调整种养结构，推行“一水三养”、“一地四收”、种养结合等循环发展模式，泥鳅鱼养殖达到7.1万亩，碱地系列蔬菜快速发展。成功举办大米河蟹北京展销会，携手央视拍摄《稻米之路》大型纪录片，“盘锦大米”品牌价值跃居全国大米行业榜首。战胜严重旱灾，粮食产量达到115.1万吨，再创历史新高。

　　优化资源配置。在严守耕地红线的基础上，通过土地集中收储、盘活存量、差别化供给，收紧房地产供地闸门，有力支持了创新创业和结构调整，提高了土地利用效益。

　　加快优化出口结构。积极开展国际产能和装备制造合作，着力推进出口基地建设，借助军贸央企开拓国际市场，地产品国际竞争力不断增强，实现出口3.42亿美元。

　　（四）鼓励创新创业，加快转换发展动能。我们着眼培育发展新动力，以小巨人企业、楼宇经济为主攻方向，推进大众创业、万众创新。培育小巨人企业30家，发展商务楼宇30.7万平方米。

　　加强产学研协同创新。大连理工大学科技园、研究院加快建设，组建3个省级专业技术创新平台、2个省级技术创新战略联盟。承接中关村产业转移，引进科技企业30余家、科技人才450余人。无人机、新材料等战略性新兴产业加快发展。

　　推进创新创业平台建设。建成6个小微企业创业园、8个创业孵化基地、5家科技孵化器，一批众创空间、创客中心蓬勃兴起。设立清华启迪天使基金等创投基金。盘锦成为全国知识产权质押融资试点市。

　　强化政策服务支撑。相继出台融资担保、减税降费等18项保障措施，加强基础配套和创业服务，有力推动了全民创新创业。新设立小微企业1.5万户，同比增长17.1%。

　　（五）推进宜居乡村建设，拓展转型发展新空间。我们把宜居乡村建设作为农村城镇化、城乡一体化的重要牵动，坚持城乡一体化规划、建设、管理与发展，不断丰富拓展宜居乡村建设内涵，所有行政村全部建成美丽村，所有建制镇全部达到示范镇标准，盘锦成为全省乃至全国城乡统筹发展的新典范。

　　全面改善农村环境。大规模推进村屯绿化、美化、亮化，历史性地实现了黑色路面户户通。引导群众加强庭院治理，建设美丽家园日益成为人民群众的自觉行动。

　　推进公共服务向农村延伸。实现燃气工程进村入户全覆盖。在全国率先建成城乡一体的垃圾集收运处理体系、市县镇村四位一体的公共卫生服务体系。完成城乡通信一体化改造，启动实施农村24小时供水改造工程，推进城乡客运公交一体化，每村建成一个文化广场，提升标准化超市、大众浴池服务能力，城乡公共服务均等化加快实现。

　　建立多元化投入、常态化管理机制，组建市县镇三级常设管理机构和村屯管理队伍，出台21项长效管理制度，宜居乡村建设进入法制化、规范化、可持续的发展轨道。

　　强化产业支撑，综合开发农村生态、社会、文化等功能，完善镇村服务业，休闲观光、民宿养老、果蔬采摘等特色村屯发展到20个。广大农村成为旅游休闲养生的好去处，既带动了农民增收，又促进了民宅增值。

　　启动宜居乡村升级版专项行动，推动宜居乡村建设向更高水平迈进，群众生活品质和幸福指数不断攀升。

　　各位代表：经过不懈努力，盘锦宜居乡村建设取得了省内外瞩目的突出成就，农村面貌发生巨大变化，为创新创业创造了广阔空间，得到人民群众的广泛支持。我们一定要坚定不移地抓下去，让城乡一体化发展的美好愿景在盘锦大地早日变成现实。

　　（六）深入推进各项改革，激发经济社会发展活力。我们围绕处理好政府和市场关系这个核心，加快建设与市场完全对接、充满活力的体制机制。

　　大力简政放权、放管结合、优化服务，取消和下放965项行政职权，取消非行政许可审批事项，市级审批事项减少80%以上。公布市政府部门权责清单。精简审批流程，全市50%的审批事项实现网上审批。在全省率先推行三证合一、一照一码、一址多照、一照多址商事制度改革。推动政府职能向事中事后监管转变，加强诚信盘锦建设，市场经济环境不断优化。

　　推进财政体制改革，出台加强财政预算资金与专项资金管理办法，硬化预算约束，压减一般性支出，提高资金使用效益。全市“三公”经费支出同比下降10.2%。

　　加强资本运作。推进国有资源资产整合和债务重组。积极争取地方政府置换债券、专项债券，以长周期低利率资金置换到期债务，政府债务风险得到有效管控。率先在全省设立10亿元市级产业（创业）投资引导基金，积极引导吸纳社会资本设立各类股权投资基金，为结构调整和创新创业创造了有利条件。广泛推进PPP项目合作，纳入国家和省项目库的项目数量和投资规模均位居全省前列。

　　开展国有农苇场市场化改革试点工作，完成海洋渔业管理体制改革，有序推进机关事业单位、国企、价格等各项改革，发展活力加快释放。

　　（七）全面推进小康社会建设，不断提高人民群众获得感。我们坚持问题导向，以民生工程、辽河口攻坚、精准扶贫、生态文明建设为突破口，扎实推进各项工作，全面建成了城乡、人头、感受上的小康社会。

　　在财政收支矛盾突出的情况下，财政用于民生领域的支出比重达到72.9%，60件民生实事全部兑现。

　　实施辽河口攻坚计划，特色产业、基础设施、公共服务体系等10个方面28项攻坚任务全面完成，经济区发生了巨大变化，得到了广大干部群众的一致拥护和高度认可。

　　扎实推进精准脱贫，以低保牢牢兜住贫困底线，建立动态管理机制，实现贫困人口动态为零。城区、县域低保标准分别提高7%和10%，继续走在全省前列。改造城市棚户区3.3万户、农垦危房2万户，完成70万平方米“暖房子”工程。

积极扩大就业，实现实名制就业4.8万人，零就业家庭保持动态为零。加快城乡居民基本医疗保险一体化，近10万农村居民享受到城镇居民医保待遇。企业退休人员养老金月人均提高183元，城乡居民基础养老金月人均提高到105元。城乡居民基本医疗保险财政补助标准提高到380元。全面实施新农合和大病保险一站式报销服务，率先在全省实现新农合报销村级即时结算。盘锦成为省社会保险城乡统筹发展试点市。

加强生态环境保护。大力推进辽河口8.6万亩退养还滩工程。实施蓝天工程，积极治理雾霾，空气质量持续改善。实施碧水工程，市第三污水处理厂如期竣工，辽河流域水质稳定达标。多措并举整治农村面源污染。全面完成省政府下达的节能减排控制目标。环境保护工作综合排名全省第一。

　　深入开展城市精细化建设与管理，创建全国文明城市工作在省检测评中荣获第一名。启动“智慧城市”建设，盘锦成为“宽带中国”示范城市。

完成一镇一所公办标准化幼儿园建设，严格执行义务教育划片招生、就近入学制度，职业教育向集团化办学迈进，盘锦大连理工大学在校学生达到4300人，形成了从学前教育到高等教育的完备教育体系。县级公立医院改革实现全覆盖。盛京医院辽东湾分院加快发展，市中心医院辽河口分院开诊运行。出生缺陷干预工程取得明显成效。

辽河文化产业园、江南风情园晋升为全国百强文化产业园区，市文化中心基本建成，我市跻身国家公共文化服务体系示范区创建行列。高水平承办中超联赛等重要赛事，成功举办市第七届运动会，群众性体育活动蓬勃发展。

坚持安全稳定常态化管理。加大隐患排查整治力度，安全生产形势总体稳定，食品药品安全稳步提升。社会治安防控体系建设深入推进，一系列大案要案快速侦破，应急处突能力不断增强。信访工作继续保持全省第一。社会大局更加和谐稳定。

　　国防动员、边海防建设、人防工作不断加强，全国双拥模范城创建再创佳绩。民族宗教、外事侨务、气象地震、统计档案、妇女儿童、残疾人等各项工作取得新成绩。

　　各位代表：经国家重点监测机构综合评估，我市无论在数据上，还是在质量上，均达到全面建成小康社会的标准。这是我们实现市第六次党代会奋斗目标的重大成果，也是我们谋划未来、奋力前行的重要基础。我们要坚定自信，在新的历史起点上，向更高的目标迈进。

　　一年来，我们深入开展“三严三实”专题教育，实施新一轮机关提效行动，政府作风加快转变，服务水平不断提高。

　　全面落实“四个着力”总要求。围绕贯彻“四个着力”、 “四个驱动、六个新的经济增长点”和市委“1+5”实施意见，科学编制“十三五”规划，制定实施13个专项行动计划和5个配套政策，加快推动“四个着力”在盘锦落地生根、开花结果。

　　推进法治政府建设。严格执行重大行政决策规则，政府决策科学化、民主化、法治化水平明显提高。自觉接受市人大及其常委会的法律监督、工作监督和市政协的民主监督，人大代表建议、政协提案办复率、满意率均为100%。大力推进政务公开，严格依法行政。圆满完成“六五”普法规划任务。

　　推进廉洁政府建设。全面加强党风廉政建设责任制和反腐败制度建设，不断加大监察审计力度，严肃查处违纪违法行为，政府廉政水平全面提高。

　　推进效能政府建设。各级干部带头走进企业、走进群众，直面矛盾、破解难题，五加二、白加黑地奋战，用时间和效率赢得了有质量、有效益、实实在在的发展。

　　各位代表：2015年的胜利收官，为我市“十二五”划上了圆满句号。五年来，在市委的坚强领导下，我们始终把盘锦放在全省全国乃至世界发展大格局中思考和谋划，围绕落实“一条主线、三大任务、四个转型”的总体布局，在实践中积极探索和创新转型之路，推动资源型城市可持续发展进入新境界。全市人均生产总值达到8.7万元，高于全省平均水平2万元以上。综合实力跃居全省第3位。

这五年，我们始终保持举全市之力开发建设辽东湾新区的战略定力，构建了“双核双城”发展格局，沿海临港布局产业带、城市带，拉开了从辽河时代挺进海洋时代的宏大序幕，奠定了盘锦参与全球竞争合作、发展更高层次开放型经济的空间基础，实现了由内陆资源型到沿海开放型的重要转变。

　　这五年，我们始终坚持以有效投资为驱动，做大做强实体经济，形成了大项目、小巨人企业、楼宇经济共同支撑转型发展的新格局，实现了由单一产业支撑到多点多元发展的重要跨越，既为我们从容布局“十三五”奠定了坚实基础，又为“十四五”实现更好更快发展积蓄了新的动能。

　　这五年，我们始终把倾力让百姓过上好日子作为不变的追求，以城乡一体化发展破解民生改善难题，先行推进城市化和城市现代化集聚辐射带动能量，以宜居乡村建设为牵动推进基础设施和公共服务向农村延伸，筑牢了民生改善之基、城乡发展之本、社会转型之实，实现了由城乡油地二元结构到城乡一体化发展的重要突破。

　　这五年，我们始终把深化改革作为转型发展的根本举措，抢抓重大机遇，争取和开展一系列国家级省级改革试点，一手抓政府职能转变，一手抓市场机制发育完善，大力推进实施简政放权、园区管理、社会治理等一揽子重大改革，使创新创业在全社会蔚然成风，生成了资源型城市转型发展的内生动力。

　　这五年，我们始终把生态文明建设作为重要的历史责任，规划建设了以生态为支撑的辽河口经济区，有效保护了湿地这一宝贵资源，全域全面推进绿色生态建设，使盘锦跻身全国生态文明先行示范区行列，既提升了城市竞争力，更保证了代际公平，走出了生态与经济共赢、湿地与城市共生的绿色发展新路。

　　各位代表：过去的五年是盘锦发展史上具有里程碑意义的重要五年。五年来，全市始终坚持一张蓝图绘到底，凝聚了万众一心谋发展促转型的强大气势，解决了一些看似不能但经过奋斗却都解决了的难事，办成了一些多年想办而没有条件办的大事，资源型城市转型发展实现了历史性跨越。实践证明，我们走对了路，干对了事，一个现代化的滨海新盘锦在渤海之滨快速崛起。

　　成绩的取得来之不易。这是市委正确领导的结果，是市人大及其常委会、市政协有效监督和大力支持的结果，更是全市人民团结奋斗的结果。在此，我代表市政府，向全市人民，向各民主党派、工商联、人民团体和无党派人士，向驻盘部队广大官兵，向辽河油田、华锦集团、辽河石化等大企业，向关心、支持盘锦发展的各界朋友，表示衷心的感谢!

　　在总结成绩的同时，我们也清醒地看到，与全国全省相同，盘锦经济发展已经进入新常态，动力转换、增速换挡的特征逐步显现。资源型城市体制机制改革还需进一步深化，市场发育不完善、对外开放度不高、主导产业链延展不够、创新动力不足、民营经济规模较小等深层次问题尚未根本解决。政府职能转变有待加强，仍然存在着行政审批前置环节多、市场监管不到位等问题，个别干部还不能完全适应经济发展新常态，抓落实的能力亟待提高。对于这些问题，我们一定高度重视，采取有力措施，认真加以解决。

　　二、“十三五”时期的目标任务

　　“十三五”时期，是盘锦全面转型的决胜期、综合优势的释放期、走出新路的关键期、全面发展的孕育期。我们要看到，盘锦仍处于可以大有可为的重要战略机遇期，但战略机遇期的内涵发生深刻变化，经济增长向更多依靠创新驱动、更多依靠内生动力、更多依靠扩大内需、更多依靠有效供给转变。

　　我们要坚持“四个全面”战略布局，牢固树立创新、协调、绿色、开放、共享的发展理念，以落实“四个着力”、实现全面转型、走向全面发展为主题，贯穿向海发展、全面转型、以港强市主线，坚守百姓增收、生态良好、社会平安三大底线，力推深化改革、做强企业、创新创业三大举措，深耕沿海开发开放、城乡一体化发展、和谐盘锦建设三大任务，依靠“四个驱动”共同发力，积极培育壮大新的增长点，切实积聚转型发展新动能，形成转型发展新支撑，打造转型发展新引擎，努力走出一条具有盘锦特色、体现规律、质量更高、效益更好、结构更优、优势充分释放的资源型城市转型发展新路，在全面建成小康社会的基础上，实现全面转型，走向全面发展，加快建设更具实力活力和竞争力的滨海新盘锦。

　　“十三五”时期的主要目标是：经济实力显著增强，地区生产总值年均增长7%以上，到2020年突破2000亿元。社会更加充满活力，跻身全国文明城市行列，城乡、油地二元结构基本消除。综合竞争力大幅提升，基本实现市域治理体系和治理能力现代化，创新成为驱动发展新引擎，主导产业迈向中高端水平。人民群众普遍幸福，城乡居民收入持续走在全省前列，成为国家级生态文明建设示范市，全市更加安全稳定和谐。

　　做好“十三五”时期工作，我们要牢牢把握“六个发展导向”，推动发展理念向全面发展转变，发展动力向混合动力转变，结构优化向竞争力转变，开放格局向资源配置转变，生产生活方式向绿色低碳转变，发展布局向城乡一体转变。扭住持续深化改革、辽东湾开发开放、做强实体经济、厚植创新创业、坚持城乡一体、强化社会治理、植根过好日子“七个战略支点”，以重点工作和关键环节的突破带动全盘、活跃全局。第一，坚持开放发展，着力完善体制机制，以辽东湾开发开放为重点打造开放发展新高地。第二，坚持协调发展，着力推进结构调整，以发展实体经济培育有竞争力的企业为重点走出协调发展新路子。第三，坚持创新发展，着力鼓励创新创业，以发展科技小巨人企业和楼宇经济为重点厚植创新发展新优势。第四，坚持绿色发展，加快推进生态文明建设，以打造生态优势、形成生态竞争力为重点开辟绿色发展新境界。第五，坚持共享发展，着力保障和改善民生，以提高人民生活质量、实现城乡一体化发展为重点创造共享发展新篇章。第六，坚持人才引领发展，加强人力资源开发建设，以营造环境、蓄积人才为重点迈出人才支撑和引领转型发展新步伐。

　　面对全市人民的期待和重托，我们一定加倍努力，奋力实现“十三五”宏伟目标，加快建成一个让全市人民普遍感到幸福、引以自豪、令人向往，宜居宜业宜游的滨海新盘锦。

　　三、2016年工作安排

　　今年是“十三五”规划的开局之年，也是推进结构性改革的攻坚之年。做好今年工作，意义重大。我们要深入贯彻市委六届十次、十一次全会精神，把“四个着力”总要求落地作为一号任务，抓好“十三五”规划实施，落实好市委“四个着力”1+5实施意见，以13个专项行动计划、5项配套政策为抓手，以供给侧结构性改革为突破口和着力点，把握好稳增长和调结构的平衡，发展实体经济，扩大有效供给，锐意改革创新，有效管控风险，持续改善民生，确保“十三五”开好局、起好步，为开启实现全面转型走向全面发展新航程全速启航。

　　今年经济社会发展主要预期目标是：地区生产总值增长6%；固定资产投资增长7%；社会消费品零售总额增长8%；一般公共预算收入增长3%；出口总额增长5%；城镇常住居民人均可支配收入增长7%；农村常住居民人均可支配收入增长9%。确保主要指标增速高于全省平均水平。

　　（一）保持经济平稳增长。稳增长仍然是今年政府工作的首要任务。我们要充分释放各类增长潜力，确保经济运行在合理区间，有效稳定市场预期。

　　全力稳定企业运行。支持企业扩产改造，抓好华锦老基地改造提升等20项技改项目，促进长春石化等企业完成后续投资。积极帮助企业降低制度性交易、税费、财务、电力、物流等各项成本，支持辽河油田、辽河石化等大企业稳产增效，夯实发展的基础。

　　保持有效投资力度。实施《全面推进项目建设专项行动计划》，实现亿元以上项目开工125项、竣工125项。确保兵器精细化工及原料工程开工建设，30万吨级原油码头获得核准。加快推进忠旺铝材全产业链基地、中储粮综合产业基地、正新轮胎、华润啤酒产业园、精细化工科技园、中蓝电子产业园、诚通纸业等重大项目。

　　突出抓好招商选资。强化常态化招商、驻地招商，着力引进投资有效益、产品有市场、企业有利润、员工有收入、政府有税收的好项目，吸引一批世界500强、隐形冠军、上市公司入驻盘锦。签约注册优质项目150项。

　　全面加强园区建设。完善基础设施，推动公共服务体系集约化发展。优化并坚持产业定位，树起鲜明的产业特色。强化集约节约用地，加大以项目为核心的考核力度，促进园区尽快摆满项目，提高土地利用强度和投入产出效益。

　　（二）加快辽东湾新区开发建设。把推进新区建设作为一号工程，举全市之力实施百项工程建设，打造落实“四个着力”的先行区，争创国家级新区，开启建区第2个10年的崭新篇章。

　　完善提升港口功能。新建续建5个5万吨级以上泊位，实施25万吨级航道工程，力争沈盘铁路全线通车、阜盘铁路开工建设，做好城市轻轨、朝盘铁路等工程前期工作并开工建设，港口吞吐能力达到6000万吨。

　　努力建设世界级产业基地。加快和运橡胶等重点项目建设，推动北燃公司原油加工能力突破800万吨，加大精细化工、特色装备、临港物流、战略性新兴产业等项目的引进力度。

　　加快创业新城建设。实施《打造辽东湾创业新城专项行动计划》。推进81项市政设施建设，抓好辽东湾海堤工程，完成综合客运枢纽主体工程。完善住宅、商业、旅游等城市功能，促进创业孵化基地产业集聚。推进粮食交易、石油化工、旅游信息三大平台建设，探索建立旅游指数、粮食交易指数发布系统，发展平台经济、指数经济、口岸经济。深化投资贸易便利化改革。把市商务中心建设成为全国一流的创新创业中心。

　　放大新区开放效应。主动融入“一带一路”、中韩自贸区和京津冀协同发展战略，争取纳入辽宁自贸试验区总体规划，推进综合保税区申报工作、做好国家进口粮食口岸报批工作，开通盘蒙欧集装箱班列。推进口岸大通关建设，提升通关便利化水平。

　　加强国际产能和装备制造合作。以新区为龙头，带动各县区扩大开放，加快出口基地建设，进一步扩大与军贸央企的对接合作，做大进出口总量。

　　（三）努力建设制造强市。制造业是盘锦发展的脊梁。我们要认真落实《中国制造2025》，实施《着力推进制造业优化升级专项行动计划》，以智能制造为主攻方向推进“两化”融合，打造制造强市。

　　构建智能制造和智能服务支撑体系。围绕提升企业研发、生产、管理和服务全程智能化水平，搭建五个云服务平台，全方位多角度支持制造业提升新能力、创造新价值。

　　推进智能制造项目建设。实施智能、创新、绿色、品牌四大关键制造工程和制造服务协同发展工程。抓好40个自动化智能化提升项目，引进德国及欧美、台湾及昆山、京津冀等重点地区的高端智能制造项目，提高重点产业数字化智能化水平，推动产品向价值链高端迈进。

　　实施工业强基工程。加强创新链、产业链建设，推进石化产业向精细化工、节能环保方向发展，特色装备制造向生产服务方向转变，加快建设世界级石化及精细化工产业基地和国内领先的特色装备制造产业基地。

　　发展战略性新兴产业。高标准规划建设无人机产业园，加快发展信息技术、生物医药、新材料、新能源等新兴产业，培育新型制造体系。

　　（四）大力发展现代服务业。以提高硬件水平、规范服务软环境、提升服务品质为主要目标，实施“深化供给侧改革规范提升城区服务业发展三年行动”，促进服务业优质高效发展。

　　用国际化视野谋划发展旅游大产业。实施《发展特色旅游业专项行动计划》，以举办红海滩国际马拉松赛为牵动，建设国际重要的湿地旅游目的地。加强红海滩廊道改造、“红马”体育广场等旅游项目建设。打造国际化旅游信息平台，加强与品牌旅行社的合作，加大旅游专列、旅游包机和游轮引进力度，开拓域外境外客源市场。精心办好四季游系列主题活动，着力塑造中国盘锦冬季稻草艺术节等冬季旅游品牌，优化旅游消费环境，让盘锦旅游走向世界。

　　促进旅游业与三次产业融合发展。释放宜居乡村建设综合优势，把农村作为接待外来游客、发展旅游产业的广阔天地，大力发展乡村休闲观光、养老度假等特色旅游业，培育一批乡村旅游产品，建设一批民宿产业村。加快建设辽河文化产业园等文化示范区，开发辽河口文化系列旅游产品，塑造和推广辽河口渔家菜品牌，丰富旅游业发展内涵。

　　推动服务业转型升级。提速发展电子商务，加快商贸物流行业标准化信息化和快递物流产业园建设，促进7个省级服务业集聚区、3个重点物流园区提档升级。积极构建以大众化市场为主体，层次丰富、业态均衡，与消费需求相适应的餐饮住宿业发展新格局。推进农产品现代流通体系和肉菜流通可追溯体系建设。积极发展金融服务业，力争引进3家以上金融机构，有效对接多层次资本市场，努力完善金融体系。加快发展研发设计、商务咨询、总部经济等新型业态。

　　化解房地产库存，以深化户籍、住房制度改革扩大有效需求，鼓励房地产企业调整营销策略，继续办好大型房交会，打通供需通道，保持房地产业持续健康发展。

　　（五）积极转变农业发展方式。实施《加快转变农业发展方式构建现代农业体系专项行动计划》，走产出高效、产品安全、资源节约、环境友好的现代农业发展道路。

　　持续增强粮食生产能力。加强农业基础设施建设，创建高标准农田11.3万亩。推进现代农业示范区建设。推广绿色增产模式，提高单产水平，确保粮食丰产丰收。

　　推进农业科技创新。推广应用高性能智能化农机装备。建立“互联网+”农机服务网络平台，实现农机作业全程网络化。

　　提高农业综合效益。推动认养农业规模化发展、信息化建设，全年达到20万亩。做大做强大米、河蟹、泥鳅鱼、碱地系列蔬菜等优势产业，加强农产品品牌建设，形成结构合理、质量安全、保障有力的农产品有效供给。发展农产品加工业，培育加工型、外向型龙头企业10家。扩大优势产品出口。积极开发农业多种功能，拓展农业增效、农民增收新渠道。

　　深化农村改革。完成农村集体土地承包经营权确权登记颁证任务，发展多种形式适度规模经营，培育新型农业经营主体。

　　（六）持续推进大众创业、万众创新。创新创业是永续发展的不竭动力。我们要实施《全面促进大众创业万众创新专项行动计划》，落实《推进大众创业万众创新若干政策》和《促进市场发育完善若干政策》，建立以创新为主要引领和支撑的经济体系、发展模式，加快转换发展动力。

　　推进产学研协同创新，争取大连理工大学获批筹建省级大学科技园，支持大学研究院加快建设，新建5所产业技术研究院。发展产业创新联盟、专业技术创新平台，推动一批创新成果实现产业化。加大知识产权保护力度。

　　加强“四众”平台等创新创业载体建设。健全完善企业管理、市场营销、人力资源、法律服务、检验检测、居家服务等生产生活服务功能，落实好《建立产业创业投资引导基金促进结构调整和创新创业若干政策》，构建全方位一站式综合服务体系。落实《促进大数据开发应用若干政策》，推进数据资源开放共享，加快产业组织、商业模式、供应链、物流链创新。科技小巨人企业达到80家，楼宇经济发展到65万平方米。

　　实施《促进个体工商户转型升级为企业专项行动计划》，加大政策扶持力度，实现个转企3000户。

　　全面落实《推进人才集聚若干政策》，打造优质的创新环境、就业环境、生活环境、政务环境，加大各类人才引进培养力度，着力培养本地人才，建设人才高地。全年引进吸纳海内外创新创业型高端人才15名、急需紧缺型人才2000名、高校毕业生8000名。

　　（七）加快城乡一体化发展步伐。打造城市和宜居乡村两个升级版，创建全国城乡一体化发展先行示范区。

　　全面提升宜居乡村建设水平。实施《打造宜居乡村建设升级版专项行动计划》，强化镇区、村屯规划建设管理，打造宜居乡村示范带，建成165个示范村。

　　加强农村基础设施精细化建设，实施“大树进村、果树进院”，实现镇区、村屯道路全面硬覆盖、绿覆盖。

　　加快公共服务向农村延伸。推进大伙房水库输水配套工程建设，实现全市农村24小时供水。开通市区到示范村的客运公交线路，通行率达到100%。完善燃气入户工程，推广应用燃气壁挂炉。

　　落实县镇村三级建管责任，推进治理重心向庭院倾斜，完善村规民约，引导群众转变生活方式，提高生活品质。

　　鼓励社会力量到农村创新创业，发展镇村特色产业，让农村成为全民创新创业的汪洋大海。

　　打造城市建设管理升级版。坚持人民城市为人民，不断提升城市环境质量，加快建设盘山新县城与双台子辽河新城，促进县城与城区同步建设，推动城市紧凑集约、高效绿色发展。实施《加强网格化管理、智慧化建设专项行动计划》，建成大数据云计算处理中心、云综合管理和公众服务系统、智慧城管、智慧社管、智慧交通等行业应用系统，把信息化精细化管理延伸到城乡每一个角落。

　　围绕促进“双核双城”发展，加强城市基础设施建设。完成中新线等交通改扩建工程。实施滨海大道、管廊街等综合管廊项目。启动海绵城市示范区建设，推进平原水库、河海、湿地与城市给排水、农业灌溉系统互联互通，建设内涝储水、干旱补水、自然净化的海绵城市。

　　推进城乡协调发展。全面实施镇村街道社区改革，推动社会管理重心和服务资源下沉。促进城乡要素平等交换、合理配置和基本公共服务均等化。抓好全国文明城市创建工作，提升城乡文明程度。

　　（八）进一步深化改革。改革是激发活力、增添动力的根本举措。我们要加大重点领域改革攻坚力度，着力形成引领经济发展新常态的体制机制。

　　实施《放宽市场准入严格市场监管专项行动计划》。坚持以减为主，依法再取消一批含金量高的职权事项，全面清理不合理的前置审批要件、证明事项和与审批相关的中介服务事项。制定公布监管职责目录，推动协同监管、综合执法、信息互联共享，建设诚信盘锦，营造公平公正的营商环境。

　　实施《促进投资与服务便利化专项行动计划》。优化审批流程，确保集中审批和便民服务事项100%网上审批。加强涉企收费目录清单动态管理。推进公共服务管理标准化建设，为群众提供更加便捷高效的公共服务。

　　深入实施资本运作。推进政府融资平台转型。组建市国有资本运营公司，成立市政建设、水务等国有企业集团，推动辽东湾、辽河口国资公司集团化发展，通过划转资产债务，推进政府与债务脱钩，促进政府购买服务等公共服务市场化改革，形成“借用管还”和投融资一体化运作机制。加大债券、专项基金、政策性贷款争取力度。推广PPP项目模式，发展股权投资基金，搭建有效利用社会资本的新通道。

　　全面落实供给侧结构性改革举措。抓好大洼县国家中小城市综合改革试点，大力推进国有农场职工住宅用地流转改革，努力在撤县改区、投融资等体制机制创新方面实现突破。推进财税体制、事业单位、不动产登记、价格、公用事业、农信社等各项改革。

　　（九）加强生态建设和环境保护。改善生态环境就是发展生产力。我们要实施《生态建设专项行动计划》，为群众提供更多更优质的生态产品，实现绿色惠民、绿色强市。

　　加强湿地保护。加快推进辽河口退养还滩工程，积极开展退耕还湿。新增国家级和省级湿地公园试点各1个。

　　整治农村面源污染。从源头上控制农药化肥使用，抓好污水处理、垃圾处理、改厕、规范畜禽养殖等系列工程。

　　实施蓝天工程。坚决淘汰燃煤小锅炉，启动华润热电二期项目，提高集中供热水平。抓好大气污染治理、秸秆综合利用、绿色交通、扬尘控制等工程，有效防范雾霾天气发生。

　　实施碧水工程。加强水源地保护。抓好一批城市污水处理厂建设项目，确保17个乡镇污水处理厂平稳运行。推广村级小型污水处理设备。实施螃蟹沟（六零河）综合整治、清水河生态治理工程，打造新的景观带、旅游带。

　　推进绿色清洁生产。加大环保执法力度，清理未批先建项目。抓好重点领域节能改造，确保完成节能减排控制目标。

　　各位代表：盘锦已经进入国家生态文明先行示范区行列。我们要实行最严格的环境保护制度，严肃查处各类环境违法行为，全力保护好盘锦的一片蓝天、一方净土、一湾绿水。

　　（十）着力保障和改善民生。促进社会公平、增进人民福祉，是我们一切工作的出发点和落脚点。我们要把守住百姓增收生态良好社会平安三条底线作为一号民生，全面落实40件民生实事，大力加强社会建设，让全市人民共享改革发展成果。

　　实施更加积极的就业政策，城镇登记失业率控制在3%以内，零就业家庭保持动态为零。完善城乡社会保障体系。深入推进全民参保计划，基本社会保险覆盖率达到96%。推行城镇职工异地居住就医直接结算。

实施辽河口巩固提升工程，推动工作重心由输血向造血转变，增强经济区内生发展动力，最终实现全市同步发展。

　　推进精准扶贫，确保贫困人口动态为零。加强扶贫开发，为有劳动能力的低保人口优先提供农村保洁员等就业岗位，实现稳定脱贫。强化动态管理，保证社会公平，坚决不养懒人。全市城乡最低生活保障标准提高7%。

　　实施《加快推进棚户区改造专项行动计划》，改造棚户区1.4万户、农垦危房1.1万户。继续开展“暖房子”改造工程。

　　深化教育综合改革，促进各级各类教育协调发展。扩大普惠性民办幼儿园覆盖率。加快县域义务教育均衡发展。推进普通高中优质特色发展。着力建设具有盘锦特色的现代职业教育体系。

　　抓好医药卫生体制改革。构建分级诊疗就医新秩序。完善市县镇村公共卫生服务一体化管理体系。推进新农合管理规范化、制度化。落实全面两孩政策。

　　推进国家公共文化服务体系示范区创建工作，打造全国有影响力的文化产业基地。确保市文化中心全面竣工并交付使用。做好第13届省运会筹备工作，广泛开展全民健身活动，促进体育产业加快发展。

　　加强安全稳定风险评估。建设统一高效的应急指挥系统，提高应急处置能力。全面排查整治各类安全隐患，严防重特大安全事故发生。创建食品药品放心城市。加快构建立体化社会治安防控体系，开展常态化治安严打整治行动，实现网上网下两个战场的有效管控，保障社会安全。打造阳光信访、法制信访、责任信访，依法保障群众合法诉求。强化法治宣传教育，启动实施“七五”普法规划。

加强国防教育和后备力量建设，巩固军政军民团结。抓好国家安全、民族宗教、外事侨务、防震减灾、妇女儿童、老龄慈善、红十字会、质监物价、统计档案、地方志、公积金等各项工作。

　　各位代表：实现“十三五”宏伟蓝图，完成今年的目标任务，必须加强政府自身建设。

　　我们要依法行政，加快建设法治政府，依法全面履行职责，自觉接受市人大及其常委会的法律监督、工作监督和市政协的民主监督，加强民主协商，广泛听取各民主党派、工商联和无党派人士的意见和建议，不断提高政府工作法治化、规范化水平。

　　我们要勤政为民，始终把群众安危冷暖放在心上，真诚倾听群众呼声，真心关注群众疾苦，以务实高效的工作作风，全心全意为人民谋福祉。

　　我们要廉洁自律，保持对纪律和规矩的敬畏，大力推进廉政建设，加强行政监察、审计监督，维护良好的政治生态和从政环境。

　　我们要真抓实干，在市委的正确领导下，市政府全力以赴抓落实，将市委总揽全局、加强顶层设计作出的决策部署和全市人民的期盼转化为具体操作方案，制定时间表、路线图，完善以项目制方式抓落实的工作制度，把激发县区活力作为一号课题，加大定量考核力度，全面提高各级政府的执行力。

　　各位代表：盘锦已经站在新的历史起点上，正在朝着实现全面转型、走向全面发展的目标迈进。让我们在市委的坚强领导下，团结一心，拼搏进取，为建设更具实力活力和竞争力的滨海新盘锦而努力奋斗！
